# Disability, Human Resources and Behavioral Economics: The Labour Inclusion Case of Ilunion Hotels of the Costa del Sol (Spain)

**DOI:** 10.3390/ijerph18157932

**Published:** 2021-07-27

**Authors:** Marco Antonio Cruz-Morato, Carmen Dueñas-Zambrana, Josefa García-Mestanza

**Affiliations:** 1 Department of Economics and Business Administration, University of Málaga, Campus El Ejido s/n, 29071 Málaga, Spain; jgm@uma.es; 2 Faculty of Social and Labour Studies, University of Málaga, Avenida Francisco Trujillo Villanueva, nº 1, 29170 Málaga, Spain; cdz@uma.es

**Keywords:** disability, human resources, behavioral economics, social inclusion, hotel industry, labour inclusion, Special Employment Centres

## Abstract

The situation of labour inclusion of people with disabilities in Spain is still too negative, in spite of the different efforts carried out by public and private sector. Previous research points to social discrimination as one of the main causes of the situation. Ilunion Hotels is one of the most important hotel companies in Spain focused on labour inclusion of people with disabilities. The objective of this paper is to explore the social inclusion case of Ilunion Hotels of the Costa del Sol, the actions that they have developed to improve the labour integration of this collective, based on a behavioral economics theoretical model (with a high relevance of the influence of social stigma, stress theories and coping to stress responses). We look into the specific situation of two of the three hotels developed as Special Employment Centres (sheltered employment contexts defined by Spanish legislation) and the possible impact of their Support Units for Professional Activity. Case study methodology is considered the most appropriate, according to the research objective, supported by semi-structured interviews with the hotel managers. The results show that, although Special Employment Centres are effective in improving labour integration in the short term and could contribute to change the long-term social perspectives about workers with disabilities, they could be also reinforcing the social stigma existing in the ordinary market.

## 1. Introduction

There is a growing awareness about the situation of social exclusion (especially in the labour market) generally suffered by groups such as people with disabilities. According to [1] (p. 7036): “the full inclusion of people with disabilities means that all of them are provided with the same conditions to function in society, including the right to make decisions about their lives, the possibility of starting a family or non-discriminatory access to workplaces”. Thus, the situation of labour inclusion of this group in economies such as Spain seems particularly dramatic. Activity rates are significantly lower than for the rest of the population, see [2,3,4]. According to [5,6], this situation is similar in the main European countries, also related to low employment rates. Further, the problem may be getting even worse as a result of the current COVID-19 pandemic, with high impact for people with disabilities [7].

Recent research has pointed out that one of the main underlying causes of the social exclusion and higher difficulties of people with disabilities is the way that the rest of society perceives them and acts in relation to the collective [8,9]. According to [10], in some European countries it is starting to grow the idea that the negative attitudes towards people with disabilities is not only important in terms of their social and labour integration, but also because these barriers are provoking a loss of an important potential resource (in terms of human capital, innovative capacity and business productivity, see [11,12,13,14]). Therefore, labour integration of people with disabilities also benefits the whole society, because people with paid employment have more self-esteem, they will carry out a full participation in society and they can start personal projects which contributes economically [15]. In addition, fighting against social exclusion also has a positive impact on economic growth itself (and not only in terms of greater equity or social justice, see [16]).

This way, it seems to be necessary to focus on this underlaying discrimination (and the way people with disabilities face it), as well as possible strategies to overcome the situation. Any initiative to change behaviours in an effective manner implicitly assumes the need to integrate Psychology into the model (see [17] or [18]). Thus, it is necessary to mention behavioral economics, which, according to [19] is the field of knowledge that studies the economic behaviour of human economic agents which involves economic decisions, as well as its determinants and consequences.

Some countries, such as Spain, have carried out legal initiatives and incentives to promote this labour inclusion, so people with disabilities can choose between regular employment (the same conditions and standards than workers without disabilities) and sheltered employment [20,21]. Specific work centres have been created to spread protected employment of people with disabilities (Special Employment Centres), and they are responsible for a high employment rate of this group of people [22,23]. These centres are born with the social objective of the labour inclusion of the collective, but the possibilities to access ordinary employment are low [23,24,25,26]. Therefore, although they are an easy and effective way to solve the situation in the short term (hiring people with disabilities), paradoxically they can be reinforcing the social stigma which they are trying to face, causing a situation of more vulnerability [23,26].

These Special Employment Centres incorporate the creation of Support Units for Professional Activity. These units are multi-professional teams, that carry out different functions and tasks, in order to overcome the barriers and possible difficulties that people with disabilities have in the process of incorporation to a job in these centres, as well as their permanence on it [27]. This figure is somehow similar to the concept of the ambassador of people with disabilities in the workplace [1], although according to previous research, the presence of this person is especially relevant to companies which had not previously employed people with disabilities.

The tourism industry has become one of the most important economic sectors in recent years, Spain being one of the most well-established and popular tourist destinations [28,29]. Although the COVID-19 crisis has also challenged the strength of the sector, in regions such as the Costa del Sol (Malaga, Spain), tourism is still one of the main economic engines, with great importance for the hotel industry (nevertheless, it has also been reported low-quality jobs as a feature of this sector, [30]). This way, it is necessary to highlight the case of the Ilunion Hotels chain (which is one of the most important hotel companies focused on the labor integration of people with disabilities in Spain). This is especially true in the Costa del Sol, in which two of its three hotels have been developed as Special Employment Centres.

Why is still the labour situation of people with disabilities so negative in developed economies such as Spain? What impact could the stigmatization processes of people with disabilities be having on their decisions regarding the labour market and their labour situation? Are Special Employment Centres (sheltered employment contexts) a sustainable solution or are they part of the long-term problem? What actions can be carried out to effectively solve the negative situation? In this article, we are going to address these issues through the following case study methodology.

This paper aims to delve into the social inclusion case of Ilunion Hotels of the Costa del Sol, the characteristics and actions that they have developed in order to boost the integration of people with disabilities into the labour market. Based on a behavioral economics theoretical model (with a high relevance of the influence of social discrimination or stigma, stress theories and coping to stress responses), we focus on the specific situation of two of the three hotels developed as Special Employment Centres and the possible impact of their Support Units for Professional Activity. The research hypothesis is that, although Special Employment Centres are effective and tempting in the short term to improve the labour integration of people with disabilities, they can be reinforcing the social stigma which they are trying to overcome (according with [23,26]). Further, the case study of Ilunion Hotels of the Costa del Sol can shed some light to it, as far as two of its three hotels have been developed as Special Employment Centres as mentioned before. 

Thus, the second section of this article briefly presents the behavioral economics theoretical model which could be explaining the situation (based on [4,31]) and delves into Special Employment Centres and their Support Units for Professional Activity, as similar to the concept of the ambassador of people with disabilities in the workplace [1]. In Section 3, methodology is described, that is to say, we used case study, content analysis and semi-structured interviews with the hotels managers (following the same procedure such as [10], asking the managers of the organizations, supported by an online questionnaire based on the questionnaire used by [1]). The next section presents the results and implications of the empirical study. Finally, the discussion, conclusions and recommendations for future research are highlighted. 

## 2. Theoretical Framework

### 2.1. Disability, Labour Inclusion and Social Stigma: A Behavioral Economics Approach 

What can be understood by “disability”? With no intention to be exhaustive, the concept of “disability” can be defined as an inability or limitation to participate in the normal life activities, caused by physical, intellectual, cognitive, sensory, emotional, psychiatric or psychological problems [23,32,33]. In Article 1, the United Nations Convention on the Rights of Persons with Disabilities [34] says that: “Persons with disabilities include those who have long-term physical, mental, intellectual or sensory impairments which in interaction with various barriers may hinder full and effective participation in society on an equal basis with others” [10]. 

People with disabilities problems and their relationship with social exclusion, labour market and human resources are issues that have been increasingly studied in recent years, highlighting the work of [1,7,10,23,35,36] or [37], among others.

As it was previously pointed out, people with disabilities are suffering a social stigma, based on discriminatory thoughts and behaviours of the rest of population, which pose a huge barrier to their labour integration [10,23,38,39,40,41]. Negative attitudes and stereotypes of hiring managers, supervisors or colleagues and other workplace conflicts, would be taking place because of the historical stigmatization, prejudice and discrimination processes of people with disabilities in the labour market [10,42]. Thus, regarding labour exclusion and discrimination of people with disabilities, it is essential to include psychology into the model (in a context of behavioral economics, as it was previously said). 

Too often, people are considered perfectly rational and cold in making decisions: the Homo Economicus prototype of traditional neoclassical economic theory [17]. However, sometimes this is not enough. It is necessary to remember that behavioral economics is the field of knowledge that completes the traditional study of the economic behaviour of human economic agents (which involves economic decisions) including more exhaustive explanations of psychological events, as well as its determinants and consequences [19]. This field is especially relevant since the publication of “*Nudge*” [43], which defined behavioral economics as “nudging”. This is important because it allows to delve into society behaviours (people with disabilities and the rest of the population, who could be developing, consciously or not, discrimination/stigmatization processes towards this group) and to boost the labour integration of this collective in an effective manner.

As [44] concluded, we could think about the psychological effects of discrimination (or stigmatization, authors such as [45], use these concepts in the same way) in people subjected to these discrimination/stigmatization processes. Very briefly, stigma can be defined as a special attribute that a person has, causing a wide discredit in other people, relegating this person to a social role different from normality [42]. In [46], the most common reasons for stigmatizing other people can be found, and among them, the justification of a system based on inequality stands out. Thus, people who stigmatize could think, consciously or unconsciously, that the stigmatized people deserve this situation.

According to authors such as [47], the fact of having a social stigma is a potential source of psychological stress. This psychological stress could lead the stigmatized person (the people with disabilities in this research) to prefer not to participate in economic activities and the labour market. In accordance with [48] (p. 1736), quoting [49]: “stigma leads some to reduce their exposure to discrimination from the larger community by restricting their activities in it”.

This way, from a psychological point of view, people with disabilities could be suffering from psychological stress. However, whether they decide (consciously or unconsciously) to participate or not in the labour market (that is to say, the fact of being hired and trying to get an employment) depends on how they can (or want) face the previously mentioned psychological stress. 

Nevertheless, if we really believe in individual freedom, perhaps it could be reconsidered to give so much importance to exogenous factors (stigmatization processes) in the choice of individual decisions. However, in practice, it is necessary to recognize the cognitive limitations that, as [50] said, some circumstances could introduce; as well as all kinds of conditioning that could affect the human will. 

In [51], it is carried out a complete classification of possible coping strategies. To sum up, these strategies could be positive (also defined as functional) or negative (dysfunctional). Most of these possible coping strategies are dysfunctional, finding not participating in economic activities to be the less risky (and more tempting) behaviour. Nevertheless, functional coping strategies also exist and some people with disabilities opt to participate in the labour market, usually related to a higher human capital and other resources provision (that is to say, a higher empowerment for this group).

Thus, [52] defend the importance of empowerment and education, in addition to the emotional cost generated by the situation of social exclusion and explain how self-esteem is damaged, especially in those with low levels of literacy.

According to [53], in line with what has been previously said, there would be two options to manage social stigma: remain “stagnant” or face the problem. However, the issue is that negative social stigmatization also aggravates other deprivations such as the lack of education or the absence of stable employment that could have been achieved through a higher educational level [53]. In this way, the process is fed back, since the importance of human capital in empowerment has been mentioned to develop functional strategies to cope with the stress caused by social stigma, and thus overcome the situation. 

On the other hand, regarding the analysis of the psychological aspects of the rest of the economic agents (consumers and companies), beyond the stigmatization processes (which, voluntarily or involuntarily, they could be developing against the group of people with disabilities), it is worth mentioning other types of different actions that could be taking place. We should not forget a wide range of actions that some people also carry out from generosity and philanthropy, such as charity, volunteering, etc., not always on an individual level, but also developed by organizations (for instance, corporate social responsibility or corporate social marketing). These actions can improve the empowerment of excluded people (such as the group of people with disabilities), awakening their participation in the market (such as potential clients or job providers, undertaking entrepreneurial actions, etc.), thus, improving their labour inclusion.

### 2.2. Special Employment Centres and Support Units for Professional Activity in Spain

As it has been said in the introduction, in countries such as Spain, there are two different ways for people with disabilities to get into labour market. On the one hand, people with disabilities can choose to get a job in the regular market, with the same characteristics and conditions than people with no disabilities. However, on the other hand, people with disabilities can also achieve a sheltered employment, protected by the State. Different legal incentives and initiatives have been developed to promote the labour inclusion of people with disabilities [20,21]. Thus, Special Employment Centres have been created as specific and sheltered work contexts. They can be developed by public or private initiatives. Special Employment Centres are created with the social objective of the labour inclusion of this group of population, and they are responsible for most of the employment of people with disabilities [22,23]. 

The social purpose of the Special Employment Centres is not only the incorporation of people with disabilities to the labour market, but there is also concerned to promote access to a quality job, with possibilities both professional and personal development [54]. However, the reality is that the type of work on these centres is generally low-skilled, the remuneration is not high, and the possibilities to access ordinary employment are also low [23,24,25,26]. Further, these are not the best indicators. 

According to [54], Special Employment Centres were created by the Law of Social Integration of Disabled People in Spain as a transit mechanism towards ordinary employment (although those workers who have difficulty integrating into the ordinary or regular market can carry out their activity in a stable way in these sheltered employment centres). The characteristic features of these centres would be [55]: carrying out productive work, regular participation in market operations, securing paid employment, provision of personal and social adjustment services, staff made up of people with disabilities (at least 70% of the workforce) and a means of integration into the ordinary labour market.

However, as it has been pointed out, the reality today is not like this. In the majority of cases, Special Employment Centres do not constitute an element of transition to the ordinary market. Rather, they are themselves an instrument for the integration of people with disabilities in the labour market. Thus, these centres could be reinforcing the social stigma suffered by people with disabilities in the labour market [23,26], although in the short term they are an effective strategy to improve the labour integration of this collective. 

Why are these centres failing to become an element of transition to the ordinary or regular labour market? Coming back to the behavioral economics theoretical framework previously exposed, it seems that this is not a measure which is contributing to modify discriminatory behaviors towards the group, but rather the opposite. Further elements should be taking into consideration in order to change discrimination behaviours in society (as well as coping responses of people with disabilities) and improve labour integration of this group in a sustainable manner. The message that seems to be remaining in society is that people with disabilities are different from the rest, instead of going in depth in the social stigma and its causes, to eliminate the differences between groups (people with disabilities and people without disabilities) existing in mental perceptions.

As it has been said, in addition to incentives and financial aid to create employment for people with disabilities (see [27,54]), Special Employment Centres incorporate the creation of Support Units for Professional Activity. Among the provision of personal and social adjustment services, these units provide workers with disabilities with greater personal rehabilitation and a better adaptation in their social relationship [55]. 

Furthermore, these Support Units for Professional Activity are usually teams formed by different professionals (the number of technical staff depends on the total amount of people with disabilities working on the centre). Their principal objective is to adapt and overcome the main barriers, obstacles and difficulties that workers with disabilities use to have in the process of incorporation to an employment in these companies (and also the problems during their permanence in the centre, see [27,54]). The functions of these units are: (a) identify those specific support needs for each disabled worker; (b) establish the necessary contacts with the environment or the family of the worker on those occasions that are required; (c) develop training programs for workers; (d) provide individualized support; (e) promote the independence and autonomy of the worker in his/her position of work; (f) facilitate the incorporation of new workers with disabilities; (g) support the worker in the transition to the ordinary market; and (h) detect the possible evolutionary deterioration of the workers to mitigate its effects. 

To conclude this section, it is necessary to highlight that the technical staff of Support Units for Professional Activity seem to share certain characteristics with the figure of the ambassador of people with disabilities in the workplace [1]. These authors define this ambassador role as a friendly person who creates a positive atmosphere about disability and workers with disabilities, trying to adapt people with disabilities to the labour context, with a high conviction of their potential. However, the main difference with the technical staff of Support Units for Professional Activity is that this figure is focused on the open labour market, and it is especially important in organizations that are not used to hire workers with disabilities. According to [1], this role is more appropriated for a middle-aged woman with knowledge about disability. It is also said that this figure reduces in a significant manner the risk of a worker with disabilities of being disapproved by his/her co-workers, helping to send them the message that the worker with disabilities is another member of the work team, with no “privileges”. This is more consistent with the behavioral economics theoretical approach developed and could really help to change discriminatory behaviors.

## 3. Materials and Methods

In this section, the methodology carried out in this research is going to be described in detail. It should be remembered that the objective of this paper is to delve into the social inclusion case of Ilunion Hotels of the Costa del Sol, the characteristics and actions that they have developed in order to boost the labour integration of people with disabilities, based on a behavioral economics theoretical model, looking at the specific situation of two of the three hotels developed as Special Employment Centres. Our research hypothesis is that Special Employment Centres can be reinforcing the social stigma which they are trying to overcome, although they are effective (and tempting) in the short term to increase the labour integration of people with disabilities.

Thus, as it was previously said, the methods implemented are case study, content analysis and semi-structured interviews with the Ilunion Hotels Managers of the Costa del Sol (Málaga, Spain) made in June 2021, supported by an online questionnaire based on the questionnaire used by [1]. It seems convenient to clarify that, although several different groups of people could be interviewed in these hotels, we have focused on the managers of the organizations (as authors such as [10] did) because they are the main decision makers, and they have a general view and the information necessary to develop this case study. Furthermore, it is important to remark that the content analysis (about company information, state of the art or press news) and the semi-structured interviews developed are useful instruments for collecting the data necessary to the case study itself as a research methodology. 

In this sense, case study would be a useful methodology to carry on with our research, according to [56], in order to verify, with a relevant practical experience, what has been hypothesized about labour inclusion of people with disabilities. Because, as it has been pointed out, a fresh perspective is necessary to address the topic [56]. Thus, the analysis of these selected cases would shed some light into the issue, and it would help to complete the theoretical framework through an inductive process in an exploratory manner. [56] proposed the following process of building theory from case study research, in a general way: (a) getting started (definition of research question or possibly a priori constructs); (b) selecting cases (specified population and theoretical, not random, sampling); (c) crafting instruments and protocols (multiplate data collection methods, qualitative and quantitative data combined, multiple investigators); (d) entering the field (overlap data collection and analysis, including field notes, flexible and opportunistic data collection methods); (e) analyzing data (cross-case pattern search using divergent techniques, for instance); (f) shaping hypotheses (iterative tabulation of evidence for each construct or search evidence for “why” behind relationships); (g) enfolding literature (comparison with similar and conflicting literature); and (h) reaching closure (theoretical saturation when possible).

The semi-structured interviews focused on the Ilunion Hotels of the cities of Málaga, Fuengirola and Mijas. That is to say, the three Ilunion Hotels of the Costa del Sol (Málaga, Andalusia, Spain). They were carried out during the month of June 2021 (specifically on 11 June 2021), conducted separately by the same researcher, member of these research team. The hotel managers were contacted for these interviews once the interest in participating on this study was confirmed with the communication managers of the Ilunion Hotels chain. To present the results in this article, the hotels would be anonymised, coded as Hotel 1, Hotel 2 and Hotel 3. As long as all data were collected by the same person and a relatively small number of participants was involved, data codification and analysis were carried out with the assistance of usual computer software analysis. 

As it has been pointed out, the interviews were supported by an online questionnaire based on the questionnaire used by [1]. The complete questionnaire used can be found in Appendix A. The main characteristics of the respondents (sex and age profile) are collected in Table 1. All the hotel managers interviewed were between 35 and 49 years old, and 67% were women. This is very important, since the managerial profile and leadership style, as we would see in next section, are very modern and proactive, as often happens in young managers (although this is not always the case).

## 4. Results

### 4.1. General Information about Ilunion Hotels 

The Ilunion Hotels chain belongs to the ONCE Social Group (one of the main organizations of people with disabilities in Spain). It has its origin in 1988, and it was born with the aim of promoting inclusive tourism, as well as the labor inclusion of people with disabilities. With 28 hotels (both urban and holiday hotels) distributed throughout the main cities of the Spanish geography, the company offers a product with inclusive design and equal treatment, through the standardization and integration of workers and also customers with disabilities. Ilunion Hotels is positioned at number 70 in the ranking of European companies “Leaders of Diversity” out of a total of 850 companies, being the first in its sector [57].

As mentioned, it dates back to 1988, when ONCE began its journey in the hotel sector. In 1993 the Confortel brand was created with the aim of unifying the image of the hotels and having a greater force. Although in 2004 they redesigned the brand to give it more strength and became Confortel Hotels. Finally, in 2015, this hotel chain was renamed again, this time as Ilunion Hotels, taking part of the conglomerate of companies that under the Ilunion brand includes all the ONCE companies and its Foundation [58].

The mission of this hotel chain is to offer customers an excellent, innovative and sustainable experience, with the commitment of a unique human team. Its vision is to continue proving to the sector, its shareholders and society that success can be achieved through a unique and sustainable business model that combines economic and social profitability. Among the main values of the company are quality and customer orientation, labour integration of people with disabilities, integrity, openness to physical and communication barriers, excellence, commitment and responsibility with a diverse society [58].

According to the information available around the chain, its turnover amounted in 2019 to 117 million euros, with an average workforce of 1243 employees (of which 40% are people with disabilities). They currently have 12 Special Employment Centres, in which more than 70% of their employees are people with disabilities. The company is working on a global strategy to promote the transformation of its hotels into Special Employment Centres. It is also the first Spanish hotel chain with the EFQM 500+ European Seal of Excellence, the highest level of this recognition. On the other hand, they also have the universal accessibility certification (UNE 170001-2) in all their establishments and the QSostenible seal.

As member of the Ilunion group of companies, it can be commented that the general figures of said conglomerate point to more than 40% of workers with disabilities (being mainly workers with physical disabilities with 59%, followed by psychosocial disability, 14%). With 57% of workers under 45 years old, a 64% predominance of permanent contracts compared to temporary contracts (36%), being also a very equal workforce in terms of sex. However, the low percentage of people with disabilities occupying managerial or middle management positions is striking [59].

Despite the harsh health and economic situation derived from the COVID-19 crisis, Ilunion Hotels presents itself as a solid social business project driven by an excellent human team and supported by the principles and values of the ONCE Social Group. Likewise, and given the difficulty facing this great crisis, it is observed that in recent times more public-private and private-private collaboration have been sought. For example, it is worth noting the fact that Ilunion Hotels offered their facilities the Government of Spain in order to decongest health services during the first wave of the pandemic [59]. Furthermore, we should also highlight collaboration agreements at a private level, for example with the Repsol Foundation, as well as with organizations in the tourism sector such as Hosteltur. 

### 4.2. Ilunion Hotels in the Costa del Sol

Currently, on the Costa del Sol (Málaga, Andalusia, Spain), one of the most important tourist destinations in the world, there are three Ilunion Hotels. They are located in Malaga city, Mijas and Fuengirola, as it has been discussed in previous sections.

The Ilunion Málaga Hotel is located on the Paseo Marítimo Antonio Machado, number 10; near the AVE high-speed train station. It is a four-star hotel with gym, spa, an indoor and outdoor pool, a fitness area and a wellness area. Four of the 179 rooms that the hotel has are accessible. This hotel is not categorized as a Special Employment Centre. 

The Ilunion Mijas Hotel, previously called Ilunion Hacienda del Sol, is a four-star hotel that is located on the Carretera de Mijas-Fuengirola, Km 4; 25 km from Malaga airport. This hotel (categorized as a Special Employment Centre) has an Andalusian architectural design, hacienda-cortijo style, surrounded by a natural and relaxing environment with views of the sea and the mountains (it is located between the Málaga mountains and the beaches of Fuengirola). Four of the 151 rooms that the hotel has are accessible. It is worth noting that this hotel has recently signed an agreement with the Mijas City Council to improve the employability options of people with disabilities in the town, through various actions such as the transfer of spaces or collaboration for the recruitment of workers with disabilities [60]. 

Finally, the Ilunion Fuengirola Hotel (also recognized as a Special Employment Centre) is located on the Paseo Marítimo Rey de España, number 87; next to the Nautical Club and the Fuengirola marina. It is also a four-star hotel, with 180 rooms (of which 14 are fully adapted for people with disabilities) and it is located on the beachfront, having privileged views of the Los Boliches area. 

It is also important to highlight that Ilunion Mijas and Ilunion Fuengirola Hotels, were the two first vacation-type hotels in Andalusia managed as Special Employment Centres, in 2018. They are also the ninth and tenth hotels that Ilunion Hotels chain manages as Special Employment Centres. For the certification as a Special Employment Centre, it is necessary that 70% of the workers are people with disabilities, as it has been noted above [61].

Now, we are going to detail the main results obtained with the semi-structured interviews (with the support of the questionnaire already mentioned) made to the hotel managers. It should be noted that, although this article does not develop a usual quantitative methodology, some basic descriptive statistics measures will be carried out, in order to make the visualization and interpretation of the results easier. However, it is not the intention of the present study to carry on quantitative methods. The objective in this research is exploratory. Let us recall that, as it has been detailed in methodology section (where the justification and convenience of the implemented methodology was carried out), our research methodology is the case study. Further, the semi-structured interviews (as well as content analysis) were carried out to get the information needed to implement this case study methodology, according to [56].

Table 2 shows how the group of directors of the Ilunion Hotels on the Costa del Sol interviewed make the human resources decisions regarding the hiring of staff jointly with other people in the organization. Which is also indicative of their leadership style.

**Figure 1:**
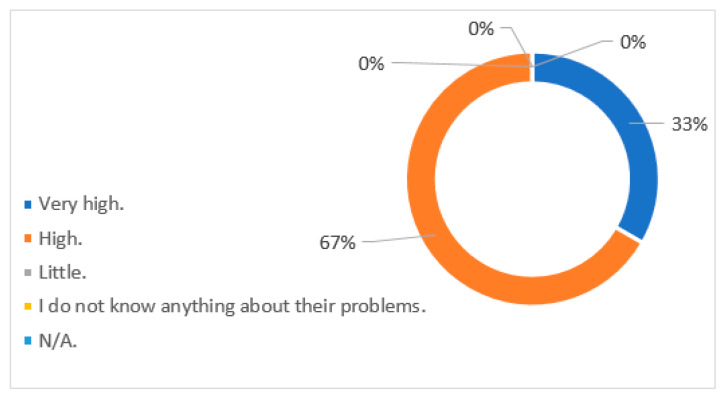
Knowledge about the difficulties of people with disabilities.

As it can be seen in Figure 1 and Table 3, these directors also show great knowledge regarding the problems of people with disabilities, having previous experience in contact with this group not only in the work context, but also in their social personal context (family and friends). It is striking, however, that none of them is a person with disabilities.

It can be seen from the Table 4 that there are certain differences between these hotels in terms of the number of staff workers (S.D. 16.09, one of the hotels having almost half the staff than others, 61–30 workers). However, it is observed that it is a workforce of a medium size, with 48 workers on average.

It should also be noted the high percentages of hiring people with disabilities, in relation to the Special Employment Centres (more than 70% in these hotels), with an average of 54%. However, significant differences are also observed with respect to centres that operate in the ordinary market (20%).

As in the case of directors, it is observed that the average age of hired workers with disabilities is low, with 100% of these workers belonging to the age segment of 30 to 49 years. This would be also possible correlated to the profile of these workers, as it will be analyzed later.

A great variety of different types of disability is observed among workers with disabilities, with 100% of the hotels highlighting the presence of workers with physical disabilities (see Table 5). However, it is also worth noting that in the hotel that has not been classified as a Special Employment Centre, the only type of disability among its workers is physical.

Although the following question (Table 6) was an open question (without predefined answer items), similar answers have been grouped. Further, it could be observed that for hotels classified as Special Employment Centres, all positions are available for people with disabilities. While for the hotel not established as a Special Employment Centre, the main positions held by people with disabilities are those of chambermaid and restaurant waiters. 

In general terms, workers with disabilities have been in the company between one and five years (100%), which makes sense considering the age profile of workers with disabilities mentioned above. Further, it also points to the relative youth of some of these centres (as well as a possible existence of staff turnover, which is also common in the tourism sector).

According to the following data (Table 7), in the selection of workers with disabilities, attitude and professional training are the most important characteristics, followed by languages (and also one of the hotels proposed the work experience). It can be seen how it would be a very similar profile to that required for a worker without disabilities in the tourism sector.

**Figure 2:**
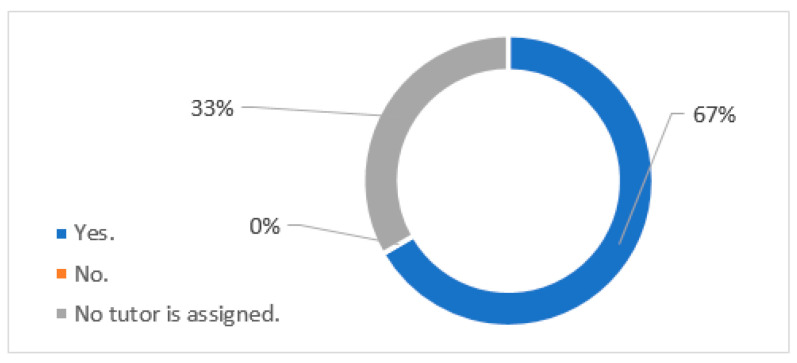
In the process of joining the company, workers with disabilities are usually assigned a tutor. Does this person also have some kind of disability?

The two Iunion Hotels rated as Special Employment Centres have technical support staff (the Support Units for Professional Activity) that also have some disabilities, according to Figure 2. The process of incorporation to the company in the hotels established as Special Employment Centres usually takes one month in a general way. No tutor is assigned in a hotel that is not a special employment centre, but, in any case, they are not obliged to have it.

As it can be seen in Table 8, all the Ilunion Hotels in the Costa del Sol agree that the limitations resulting from disabilities can be effectively compensated by a suitable workplace or equipment (the mean is 4 on a scale of 1 to 5, with 1 being “I completely disagree” and 5 “I completely agree”). However, it should be noted that one of the highest scores has been awarded in a Special Employment Centre.

**Figure 3:**
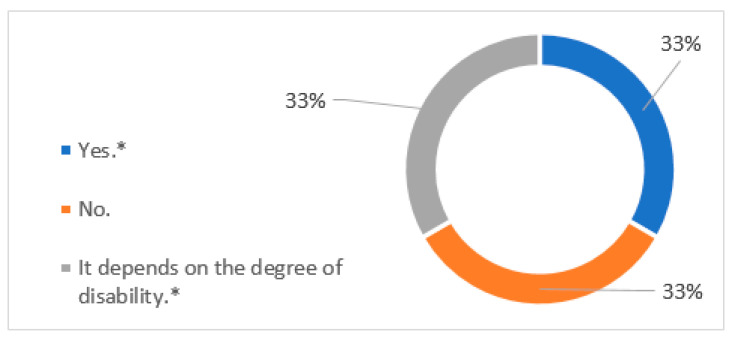
Do people with disabilities have any special circumstances with respect to other workers, for example, a shorter working day or different contracts?

As we can see in Figure 3, one hotel said that workers with disabilities have some special circumstances with respect to other workers, another hotel answered in a negative way, and the third one said that “It depends on the degree of disability”. The hotel that indicates “yes” has specified that these special circumstances are “reduced hours”. On the other hand, the hotel which said, “It depends on the degree of disability”, has indicated “adaptations to the position (loads, lighting level, movements)”. It should be noted that among these responses is the hotel that has not been classified as Special Employment Centre. 

Although this question is an open question, similar responses have been grouped together, and it can be observed in Table 9 that for the hotel not classified as a Special Employment Centre, it is also important to carry out more specific monitoring by the head of department, in addition to normalise situations.

Analyzing the following table, it can be seen that all the Ilunion Hotels in the Costa del Sol think that the level of cohesion and teamwork of the staff is very high, with an average of 4 (from 1, very low, to 5, very high). It is necessary to highlight that one of the highest scores was awarded again in a Special Employment Centre (Table 10).

Regarding the attitude/opinion that the hotel managers think that clients have, in general terms, regarding the care/treatment received of workers with disabilities, all of them agree that this would be a good opinion.

The best valuated aspects to customers, according to the director opinions, are customer support (100%), and maintenance of common areas and cleaning (67%), as it can be seen from the data in the Table 11.

Before the COVID-19 crisis, the situation of these hotels was positive, in terms of occupation levels, above 80% in all of them (an average of 84% and little standard deviation, see Table 12). However, some of the hotels classified as Special Employment Centres show the lowest percentages.

Regarding the situation during the COVID-19 pandemic, it is observed that one hotel remained closed during the first part of the crisis. The other two hotels were at 50%, with a very strong negative impact (an average of 33% of occupation rates). A greater dispersion is also observed (S.D. 0.29, it seems to have affected more in the Special Employment Centres).

Nowadays, Hotel 1 is the most affected, according to Table 13. Hotels 2 and 3 have higher occupation rates, although still far from the pre-pandemic figures (at least, all of them have recovered activity). In any case, we see less dispersion in the results than during the pandemic (although greater than before it). The strong impact trend in some Special Employment Centres is consolidated.

**Figure 4:**
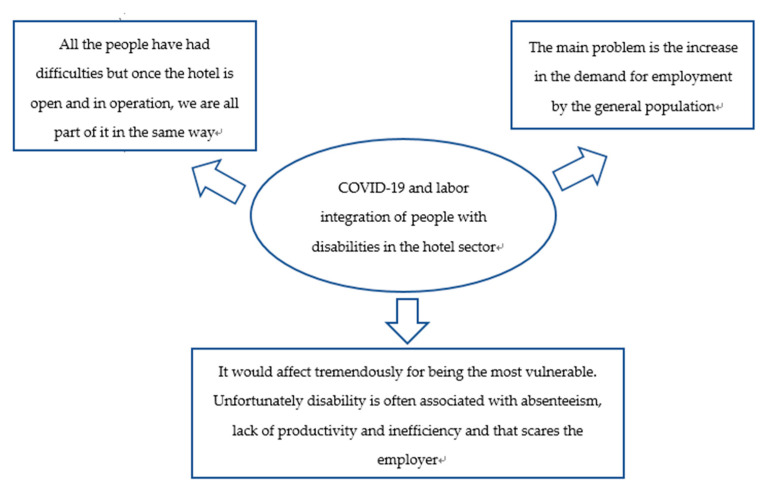
How do you think the COVID-19 crisis may be and will affect the labor integration of people with disabilities in the hotel sector?

Regarding how the directors think that the COVID-19 crisis may be affecting and will affect the labor integration of people with disabilities in the hotel sector (Figure 4), one of the interviewed managers focus on the problem of a higher demand for employment by the general population. It is also said that crisis is a general problem, it is not only for people with disabilities, but the reopening of the hotel will have a favorable impact for all of them. It is striking that one of the responses includes, even if it is to talk about it in negative terms, the social stigma on people with disabilities in the labor market, presenting a possible conception by the employers in society of the worker with disabilities as an employee with lower productivity and efficiency and higher absenteeism (ideas that should be fought against in line with the theoretical model proposed).

**Figure 5:**
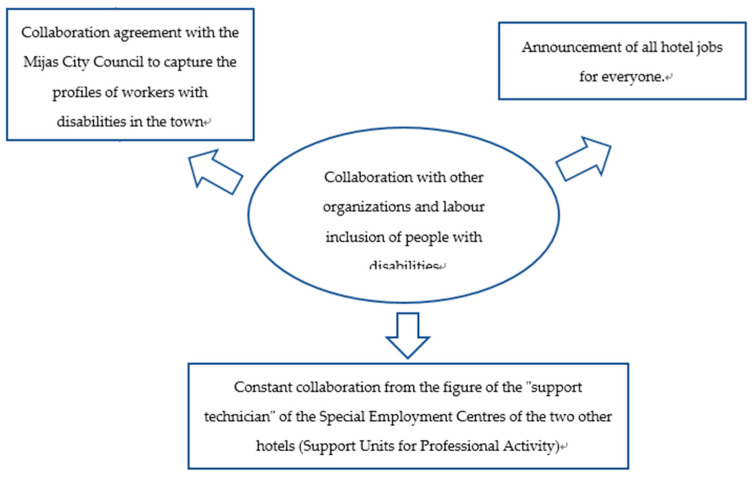
What collaboration actions are being carried out or are planned to be carried out with other organizations (public or private) regarding the labor inclusion of people with disabilities?

In relation to collaboration with other organizations to promote labor inclusion of people with disabilities (Figure 5), the agreements with public institutions stand out. It should also be explained that the hotel not classified as a Special Employment Centre receives the help of the technicians of the Special Employment Centres (Support Units for Professional Activity).

From Table 14, the following can be commented: First of all, there could be seen a strong agreement with the statement, “The competent public authorities carry out an effective policy that allows the full integration of people with disabilities in your country” (with an average of 3.33 on a scale of 1 to 4, and a standard deviation of 0.58). Regarding the second statement (“There is a social atmosphere of understanding of the needs and possibilities of people with disabilities in your country”), the average is lower (2.67, with the same standard deviation of 0.58), but the score given by the non-Special Employment Centre is even lower. The average figure regarding the statement “People with disabilities who have a job should have special employee privileges, for example, a shorter working day, longer holidays, etc., in your country” is 2, closer to the answer “definitely not” (with a higher standard deviation of 1), but, in this case, the non-Special Employment Centre has given a higher score (because they agree with that proposition). Finally, for the statement “employers in your country get sufficient knowledge on how to employ a person with disabilities and organize his/her work” the average is 3 (standard deviation 1 again), but one more time the non-Special Employment Centre has a different behavior, in this case with a lower score.

## 5. Discussion

As [23] said, it is necessary to underline the limited empirical evidence about workers with disabilities existing in the scientific literature. The present research tries to shed some light, from a behavioral economics perspective as mentioned before, on the situation of low labour integration of people with disabilities, through a case study methodology of the Ilunion Hotels of the Costa del Sol (this methodology is considered the most appropriated, according to the research objectives, see [56]).

It would be convenient to remember that our research hypothesis defends that, although Special Employment Centres are effective and tempting in the short term to improve the labour integration of people with disabilities, they can be reinforcing the social stigma which they are trying to overcome (according with [23,26]). Thus, results show that it could be happening, but it would be also necessary to carry out certain assessments.

First of all, in a general manner about the Ilunion Hotels company, the case study has shown that this chain is one of the most important companies of the hotel industry in Spain that promotes labour inclusion of people with disabilities [58,59]. In spite of the COVID-19 crisis, the company remains solid (although more public-private and private-private collaboration have been developed). Ilunion Hotels have 12 Special Employment Centres out of their 28 hotels. These centres are specific and sheltered work centres for people with disabilities regulated by the Spanish law as we have defined (see [27,54,55]), in which more than 70% of their employees are people with disabilities. Furthermore, the company is working on a strategy to promote the transformation of its hotels into Special Employment Centres.

Focusing on the three Ilunion hotels of the Costa del Sol (Málaga, Mijas and Fuengirola), the results point to the existence of two different groups in terms of their behaviour: hotels classified as Special Employment Centres and those that are not. In any case, the leadership style of the young managers of these three hotels is very modern, proactive and team oriented, with great knowledge regarding the problems of people with disabilities and having previous experience in contact with this group. This can undoubtedly help the labor insertion of people with disabilities according to our theoretical framework (although none of them is a person with disabilities).

Next, we will proceed to specify the main differences detected between these two groups, according to our theoretical framework regarding the labor inclusion of people with disabilities. Furthermore, we try to explain them from that perspective (many of the possible explanations are related to each other, so we will try not to be repetitive):

1. A high percentage of workers with disabilities work in Special Employment Centres (significant differences are observed with the non-Special Employment Centre). This reinforces the idea that in the short term, the Special Employment Centres help the labour inclusion of the collective [22,23].
2. There are different types of disability in Special Employment Centres (but only physical disabilities in the non-Special Employment Centre). Here there is a double possible interpretation: (a) given that the protected market is the “safest” alternative for people with disabilities to cope with social stigma [51], it is preferred by all of them; (b) in the regular market, the existence of discrimination is more visible, affecting in different ways depending on the type of disability.
3. All positions are available for people with disabilities in Special Employment Centres (only chambermaid and restaurant waiters are the usual positions in the non-Special Employment Centre). The possible interpretation is similar than the previous one.
4. Technical support staff (the Support Units for Professional Activity) that also have some disabilities in Special Employment Centres. There is no support staff in the non-Special Employment Centre (however, they are not obliged to have it [55]). Further, they receive the help of the technicians of the two other hotels anyway.
5. Although all of the hotels agree that the limitations resulting from disabilities can be effectively compensated by a suitable workplace or equipment, one of the highest scores has been awarded in a Special Employment Centre. Again, the explanation could be similar than the one made in the second point (perhaps stigmatizing attitudes flourish to a greater extent in the regular market).
6. No special circumstances (holidays, schedules, etc.) have the workers with disabilities in Special Employment Centres, as a general rule.
7. More specific monitoring of worker with disabilities by the head of department (in addition to normalize situations) is required in the non-Special Employment Centre.
8. Although the level of cohesion and teamwork of the staff is very high in all of them, one of the highest scores was awarded in a Special Employment Centre.
9. Regarding the occupation levels of the hotel, some of the Special Employment Centres have lower figures before the COVID-19 pandemic (although they were positive) and they have suffered the consequences of the crisis to a greater extent.
10. The non-Special Employment Centre thinks that “there is no social atmosphere of understanding of the needs and possibilities of people with disabilities in their country”, “people with disabilities who have a job should have special employee privileges, for example, a shorter working day, longer holidays, etc. in their country” and “employers in their country get no sufficient knowledge on how to employ a person with disabilities and organize his/her work”, in a different way than Special Employment Centres.

However, there are also similar patterns of behaviour between these two groups, such as the age profile of workers with disabilities (30–49 years old); the importance of attitude, professional training, languages and work experience as the most important characteristics for workers with disabilities; or the permanence in the company (between one and five years). As it was said, most of these coincidences, however, are also common in the tourism sector in general (not only for workers with disabilities).

Furthermore, it is well worth to highlight that, about how the hotel managers think that the COVID-19 crisis may (and will) affect the labor integration of people with disabilities in the hotel industry, it is striking, as it has been commented, that one of the responses includes, even if it is to talk about it in negative terms, the social stigma about people with disabilities in the labor market. Presenting a possible conception by the employer (existing in society) of the worker with disabilities as an employee with lower productivity and efficiency and higher absenteeism. According to the behavioral economics theoretical framework approach [4,31], this is the kind of social stigma that could be also provoking psychological stress on people with disabilities, which takes them away from the labour market. Therefore, it is essential to delve into sustainable strategies to enhance functional coping strategies to deal with this psychological stress and improve the labor inclusion of people with disabilities.

It is also necessary to highlight that the discussion and implications of this case study results can be taken into consideration in other different geographical contexts. We have studied a specific case in Spain, but this kind of analysis and their consequences could also be replicated in other developed economies (not only in the European Union) and also developing economies (although their starting situation may be even worse), following the Sustainable Development Goals. However, the social, cultural, labour, technological and legal context of each area should be taken into consideration for the adaptation of the model presented here to other geographical areas. Different discrimination levels, social characteristics and perceptions, or legal frameworks (sheltered employment contexts for people with disabilities), for instance, make it necessary to analyze in depth each particular case.

## 6. Conclusions

The aim of this paper has been to delve into the social inclusion case of Ilunion Hotels of the Costa del Sol (Spain), the characteristics and actions that they have developed in order to boost the integration of people with disabilities into the labour market, based on a behavioral economics theoretical model (with a high relevance of the influence of social discrimination or stigma, stress theories and coping to stress responses). Looking at the specific situation of two of the three hotels developed as Special Employment Centres and the possible impact of their Support Units for Professional Activity.

Therefore, according to the results that have been discussed in previous sections, the main conclusions are:

- The Ilunion Hotels chain is one of the most important companies of the hotel industry in Spain that promotes labour inclusion of people with disabilities.
- Some of their hotels have been qualified as Special Employment Centres (sheltered work). In the short term, these Special Employment Centres help the labour inclusion of the collective.
- Special Employment Centres can be reinforcing the social stigma which they are trying to overcome. This protected market would be the “safest” alternative for people with disabilities to cope with social stigma. Therefore, it could be preferred by people with disabilities (in case they do not prefer to cope with this social stigma in a dysfunctional manner, not participating in labor activities). However, this fact distances them from the ordinary market and deepens the social perception of the difference between people with and without disabilities.
- In the regular market, the existence of discrimination is more visible. Therefore, in the non-Special Employment Centre, labour integration of people with disabilities is even harder.
- Nevertheless, Special Employment Centres could be also contributing to change somehow the long-term social perspectives in a positive way, among clients and society as a whole, about workers with disabilities, trying to make visible that this people do not have differences to be able to carry out their work properly.
- Technical support staff (the Support Units for Professional Activity of Special Employment Centres) can be also helping to integrate this collective, trying to reduce and eliminate all kind of differences directly in the context of work, and fighting against labour stigma where it specifically occurs. As similar to the concept of the ambassador of people with disabilities in the workplace [1], this figure is even more necessary in the ordinary market.
- There are some limitations of this study and thus the possible future research suggestions are as follows: (a) to carry on a research with a higher sample of hotels; (b) to interview also workers with disabilities (not only managers); (c) to carry on some quantitative analysis; (d) to delve into the causes of social stigma and more effective ways to change behaviours (reducing/eliminating social stigma, boosting positive behaviours and enhancing functional coping strategies to deal with this psychological stress and improve the labour inclusion of people with disabilities); and (e) to analyze the situation in other geographical contexts (with similar or different social, cultural, economic, technological and legal factors).

To conclude, it is important to emphasize the relevance of developing Special Issues like this one in high impact scientific journals such as IJERPH, in order to improve the existing knowledge and overcome the limited empirical evidence about workers with disabilities of the current scientific literature mentioned by [23]. Thus, with this paper we will humbly hope to contribute to the development of this research line and lay the foundations for future research that can add value to improving the labour situation of people with disabilities.

## Figures and Tables

**Table 1:** Sex and age of the hotel managers interviewed.

Sex	Result
Man	33%
Woman	67%
Age	Result
18–34 years	0%
35–49 years	100%
50 years or more	0%

**Table 2:** Are you the person responsible for making hiring decisions in the company?

Answer	Result
Main person responsible for making hiring decisions.	0%
Making hiring decisions together with other people.	100%
Little influence on the hiring decision.	0%
Does not make decisions about hiring candidates.	0%

**Table 3:** Experience in contact with people with disabilities (check one or more).

Answer	Result
People with disabilities in my family.	33%
People with disabilities among my friends.	67%
People with disabilities in my neighborhood.	0%
People with disabilities in my workplace.	67%
I am a person with disabilities.	0%
I professionally care for people with disabilities	33%
I have no experience with people with disabilities.	0%

**Table 4:** How many people make up the staff?

Item	Result
Average staff number	48
S.D.	16.09

**Table 5:** What type or types of disability do most of your workers with disabilities have? (Check one or more answers).

Answer	Result
Physical disability.	100%
Sensory disability.	67%
Intellectual disability.	67%
Mental disability.	67%
Visceral/organic disability.	67%
Multiple disability.	67%

**Table 6:** What positions do your workers with disabilities usually occupy?

Answer	Result
All positions.	67%
Chambermaid and restaurant waiters.	33%

**Table 7:** When selecting candidates with disabilities, what profile is required (skills, training level, etc.)? (Check one or more options).

Answer	Result
Attitude.	100%
Languages.	33%
Professional training.	100%
Graduates.	0%
Master’s degree.	0%
Other: Experience	33%

**Table 8:** Do you think that the limitations resulting from disabilities can be effectively compensated by a suitable workplace or equipment? Answer on a scale of 1 to 5, with 1 being “I completely disagree” and 5 “I completely agree”.

Item	Result
Hotel 1	4
Hotel 2	3
Hotel 3	5
Mean	4
S.D.	1

**Table 9:** What policies do you carry out so that employees with disabilities feel part of the company and thus increase their productivity?

Answer	Result
Normalize situations, all workers (with disabilities or not) are equal.	100%
More specific monitoring by the head of department.	33%
Make all employees feel satisfied with their duties.	33%

**Table 10:** How would you assess the level of cohesion and teamwork of the staff? From 1 (very low) to 5 (very high).

Item	Result
Hotel 1	5
Hotel 2	4
Hotel 3	5
Mean	4.67
S.D.	1

**Table 11:** What are the best valued aspects to customers? Mark one or more options.

Answer	Result
Cleaning.	67%
Facilities.	33%
Maintenance of common areas.	67%
Quality and variety of service.	33%
Safety.	33%
Customer loyalty.	33%
Customer support.	100%
Quick service.	33%
Other:	0%

**Table 12:** What occupation average levels, as a percentage, did you have before the start of the COVID-19 pandemic?

Item	Result
Hotel 1	80%
Hotel 2	91%
Hotel 3	80%
Mean	84%
S.D.	0.06

**Table 13:** What occupation average levels, as a percentage, do you currently have?

Item	Result
Hotel 1	35%
Hotel 2	60%
Hotel 3	70%
Mean	55%
S.D.	0.18

**Table 14:** In your opinion, answer the following questions on a scale of 1 to 4, with 1 being “definitely not” and 4 being “definitely yes”.

Answer	Mean	SD
(a) The competent public authorities carry out an effective policy that allows the full integration of people with disabilities in your country.	3.33	0.58
(b) There is a social atmosphere of understanding of the needs and possibilities of people with disabilities in your country.	2.67	0.58
(c) People with disabilities who have a job should have special employee privileges, for example, a shorter working day, longer holidays, etc., in your country.	2.00	1.00
(d) Employers in your country get sufficient knowledge on how to employ a person with disabilities and organize his/her work.	3.00	1.00

## Data Availability

Restrictions apply to the availability of these data. Data was obtained from Ilunion Hotels and are available from the authors with the permission of this company.

## Appendix A. Questionnaire Used in Semi-Structured Interviews

Section 1: Participant profile.
  1.1. What is the name of the hotel where you work?
    Ilunion Málaga.
    Ilunion Mijas.
    Ilunion Fuengirola.
  1.2. What is your contact email?
  1.3. Sex:
    Man.
    Woman.
  1.4. Age:
    18–34 years.
    35–49 years.
    50 years or more.
  1.5. Are you the person responsible for making hiring decisions in the company?
    Main person responsible for making hiring decisions.
    Making hiring decisions together with other people.
    Little influence on the hiring decision.
    Does not make decisions about hiring candidates.
  1.6. Knowledge about the difficulties of people with disabilities:
    Very high.
    High.
    Little.
    I do not know anything about their problems.
    N/A.
  1.7. Experience in contact with people with disabilities (check one or more):
    People with disabilities in my family.
    People with disabilities among my friends.
    People with disabilities in my neighborhood.
    People with disabilities in my workplace.
    I am a person with disabilities.
    I professionally care for people with disabilities.
    I have no experience with people with disabilities.
Section 2: Labour inclusion of people with disabilities.
  2.1. How many people make up the staff?
  2.2. What percentage of your employees are people with disabilities?
  2.3. What is the predominant age profile of your workers with disabilities?
    18–29 years.
    30–49 years.
    50 years or more.
  2.4. What type or types of disability do most of your workers with disabilities have? Check one or more answers:
    Physical disability.
    Sensory disability.
    Intellectual disability.
    Mental disability.
    Visceral/organic disability.
    Multiple disability.
  2.5. What positions do your workers with disabilities usually occupy?
  2.6. In general terms, how many years have your workers with disabilities been in the company?
    Between 1 and 5 years.
    Between 6 and 10 years.
    Between 11 and 15 years.
    Between 16 and 20 years.
    21 years or more.
  2.7. When selecting candidates with disabilities, what profile is required (skills, training level, etc.)? (Check one or more options):
    Attitude.
    Languages.
    Professional training.
    Graduates.
    Master’s degree.
    Other:
  2.8. In the process of joining the company, workers with disabilities are usually assigned a tutor. Does this person also have some kind of disability?
    Yes.
    No.
    No tutor is assigned (go to Question 2.10).
  2.9. In relation to the previous question, how long does this process of incorporation to the company usually take?
    One month.
    From 2 to 6 months.
    More than 6 months.
  2.10. Do you think that the limitations resulting from disabilities can be effectively compensated by a suitable workplace or equipment? Answer on a scale of 1 to 5, with 1 being “I completely disagree” and 5 “I completely agree”.
    1.
    2.
    3.
    4.
    5.
  2.11. Do people with disabilities have any special circumstances with respect to other workers, for example, a shorter working day or different contracts?
    Yes.*
    No.
    It depends on the degree of disability.*
    * If in the previous question (2.11) you have chosen the option “Yes”, indicate which one/which ones. If you have chosen the option “It depends on the degree of disability”, specify what those circumstances would be:
  2.12. What policies do you carry out so that employees with disabilities feel part of the company and thus increase their productivity?
  2.13. How would you assess the level of cohesion and teamwork of the staff? From 1 (very low) to 5 (very high)
    1.
    2.
    3.
    4.
    5.
  2.14. What attitude/opinion do you think clients have, in general terms, regarding the care/treatment received of workers with disabilities?
    Good.
    Normal.
    Bad.
    N/A.
  2.15. What are the best valued aspects to customers? Mark one or more options.
    Cleaning.
    Facilities.
    Maintenance of common areas.
    Quality and variety of service.
    Safety.
    Customer loyalty.
    Customer Support.
    Quick service.Other:
  2.16. What occupation average levels, as a percentage, did you have before the start of the COVID-19 pandemic?
  2.17. What occupation average levels, in percentage, have you had during the COVID-19 pandemic?
  2.18. What occupation average levels, as a percentage, do you currently have?
  2.19. How do you think the COVID-19 crisis may be and will affect the labour integration of people with disabilities in the hotel sector?
  2.20. What collaboration actions are being carried out or are planned to be carried out with other organizations (public or private) regarding the labor inclusion of people with disabilities?
  2.21. In your opinion, answer the following questions on a scale of 1 to 4, with 1 being “definitely not” and 4 being “definitely yes”:
    The competent public authorities carry out an effective policy that allows the full integration of people with disabilities in your country.
    There is a social atmosphere of understanding of the needs and possibilities of people with disabilities in your country.
    People with disabilities who have a job should have special employee privileges, for example, a shorter working day, longer holidays, etc., in your country.
    Employers in your country get sufficient knowledge on how to employ a person with disabilities and organize his/her work.
